# Sall4 controls differentiation of pluripotent cells independently of the Nucleosome Remodelling and Deacetylation (NuRD) complex

**DOI:** 10.1242/dev.139113

**Published:** 2016-09-01

**Authors:** Anzy Miller, Meryem Ralser, Susan L. Kloet, Remco Loos, Ryuichi Nishinakamura, Paul Bertone, Michiel Vermeulen, Brian Hendrich

**Affiliations:** 1 Wellcome Trust – Medical Research Council Stem Cell Institute, University of Cambridge, Cambridge CB2 1QR, UK; 2 Department of Biochemistry, University of Cambridge, Cambridge CB2 1QR, UK; 3 Department of Molecular Biology, Faculty of Science, Radboud Institute for Molecular Life Sciences, Radboud University, 6525 GA Nijmegen, The Netherlands; 4 European Molecular Biology Laboratory (EMBL), European Bioinformatics Institute, Wellcome Trust Genome Campus, Cambridge CB10 1SD, UK; 5 Department of Kidney Development, Institute of Molecular Embryology and Genetics, Kumamoto University, Kumamoto 860-0811, Japan

**Keywords:** Sall4, NuRD, ES cells, Enhancer, Transcription factor, Co-repressor

## Abstract

Sall4 is an essential transcription factor for early mammalian development and is frequently overexpressed in cancer. Although it is reported to play an important role in embryonic stem cell (ESC) self-renewal, whether it is an essential pluripotency factor has been disputed. Here, we show that Sall4 is dispensable for mouse ESC pluripotency. Sall4 is an enhancer-binding protein that prevents precocious activation of the neural gene expression programme in ESCs but is not required for maintenance of the pluripotency gene regulatory network. Although a proportion of Sall4 protein physically associates with the Nucleosome Remodelling and Deacetylase (NuRD) complex, Sall4 neither recruits NuRD to chromatin nor influences transcription via NuRD; rather, free Sall4 protein regulates transcription independently of NuRD. We propose a model whereby enhancer binding by Sall4 and other pluripotency-associated transcription factors is responsible for maintaining the balance between transcriptional programmes in pluripotent cells.

## Abstract (precis)

**Highlighted article:** Sall4 and Sall1 inhibit neural differentiation in ESCs by acting at enhancer sequences independently of the NuRD complex, and are dispensable for the maintenance of pluripotency.

## INTRODUCTION

Embryonic stem cells (ESCs) have the potential to form any somatic cell type in the adult organism; that is, they are pluripotent. In order to properly execute lineage decisions, pluripotent cells must precisely coordinate their gene expression programmes. To successfully initiate differentiation down one particular lineage, a cell must activate the gene regulatory network (GRN) appropriate to enter that lineage, and not those corresponding to any other lineage, while also extinguishing the pluripotency GRN. It is clear from a large number of studies that the coordinated action of multiple transcription factors and chromatin-modifying proteins is essential to maintain the delicate balance between self-renewal and differentiation of ESCs (Morey et al., 2015; Niwa, 2007; Signolet and Hendrich, 2015). Although it is relatively straightforward to show that a given protein plays some role in ESC differentiation, often the precise mechanisms of how the important transcription factors function remain ill-defined.

In this study we focus on Sall1 and Sall4, the only two members of the *spalt* gene family of C2H2-type zinc-finger transcription factors that are expressed in ESCs (reviewed by de Celis and Barrio, 2009). In humans, mutations in *SALL4* show haploinsufficiency, resulting in the autosomal dominant Okihiro/Duane-Radial Ray and IVIC syndromes (Al-Baradie et al., 2002; Kohlhase et al., 2002; Sweetman and Munsterberg, 2006), while mutations in *SALL1* lead to the autosomal dominant Townes-Brocks syndrome (Kohlhase et al., 1998). *SALL4* is also aberrantly expressed in many cancers and correlates with poor prognosis, leading it to be heralded as a new cancer biomarker and potential therapeutic target (Zhang et al., 2015). In mice, Sall4 has been shown to play an essential role in peri-implantation development (Elling et al., 2006; Sakaki-Yumoto et al., 2006; Warren et al., 2007), while Sall1 is dispensable for early embryogenesis but is essential for kidney development (Kanda et al., 2014; Nishinakamura et al., 2001).

The role played by Sall4 in ESCs has been the subject of some debate. Studies using *Sall4* null ESCs concluded that it was dispensable for self-renewal of ESCs, but that mutant cells were prone to differentiate in certain conditions, indicating that it might function to stabilise the pluripotent state (Sakaki-Yumoto et al., 2006; Tsubooka et al., 2009; Yuri et al., 2009). By contrast, studies in which Sall4 was knocked down in ESCs led to the conclusion that it plays an important role in the maintenance of ESC self-renewal (Rao et al., 2010; Zhang et al., 2006). Sall4 was found to bind regulatory regions of important pluripotency genes such as of *Pou5f1* (previously known as *Oct4*) and *Nanog* (Wu et al., 2006; Zhang et al., 2006) and a physical interaction with the Pou5f1 and Nanog proteins has been reported (Pardo et al., 2010; Rao et al., 2010; van den Berg et al., 2010; Wu et al., 2006). The consensus arising from these studies was that Sall4 is instrumental in the regulation of key pluripotency genes and is thus a key regulator of the pluripotency transcriptional network (van den Berg et al., 2010; Xiong, 2014; Yang et al., 2010). Whether it is essential for self-renewal remains a point of contention.

Sall1 and Sall4 have both been shown to interact biochemically with the Nucleosome Remodelling and Deacetylase (NuRD) complex. NuRD is a transcriptional regulatory complex that has nucleosome remodelling activity due to the Chd4 helicase and protein deacetylase activity due to Hdac1 and Hdac2. Additional NuRD components are the zinc-finger proteins Gatad2a/b, SANT domain proteins Mta1/2/3, histone chaperones Rbbp4/7, structural protein Mbd3 (which can be substituted for by the methyl-CpG-binding protein Mbd2) and the small Cdk2ap1 protein (Allen et al., 2013; Le Guezennec et al., 2006). The usual interpretation of the Sall-NuRD interaction is that Sall proteins recruit NuRD to influence transcription of their target genes (Kiefer et al., 2002; Kloet et al., 2015; Lauberth and Rauchman, 2006; Lu et al., 2009; Yuri et al., 2009). The relationship between Sall proteins and NuRD might not be so straightforward, however, as they show opposing functions in ESCs. Whereas Sall1 and Sall4 are implicated in maintenance of the ESC state, NuRD functions to facilitate lineage commitment of ESCs (Kaji et al., 2006; Reynolds et al., 2012; Signolet and Hendrich, 2015).

In this study we set out to define the function of Sall4 in ESCs and to understand the relationship between NuRD and Sall4. We use defined culture conditions (2i/LIF) (Ying et al., 2008) to show that Sall1 and Sall4 prevent activation of neural genes in ESCs, but are dispensable for the maintenance of the pluripotency GRN. We further show that although NuRD is the major biochemical interactor of Sall4, only ∼10% of Sall4 protein associates with NuRD in ESCs. Despite this interaction, Sall4 neither recruits the NuRD complex to chromatin nor shows NuRD-dependent transcriptional regulation. The majority of Sall4 has no stable biochemical interactors, but colocalises with pluripotency-associated transcription factors at enhancer sequences. We propose a model to explain why accumulation of these transcription factors can stimulate the transcription of some genes but inhibit the expression of others.

## RESULTS

### Sall4 is dispensable for ESC self-renewal, but inhibits neural differentiation

**Fig. 1.**
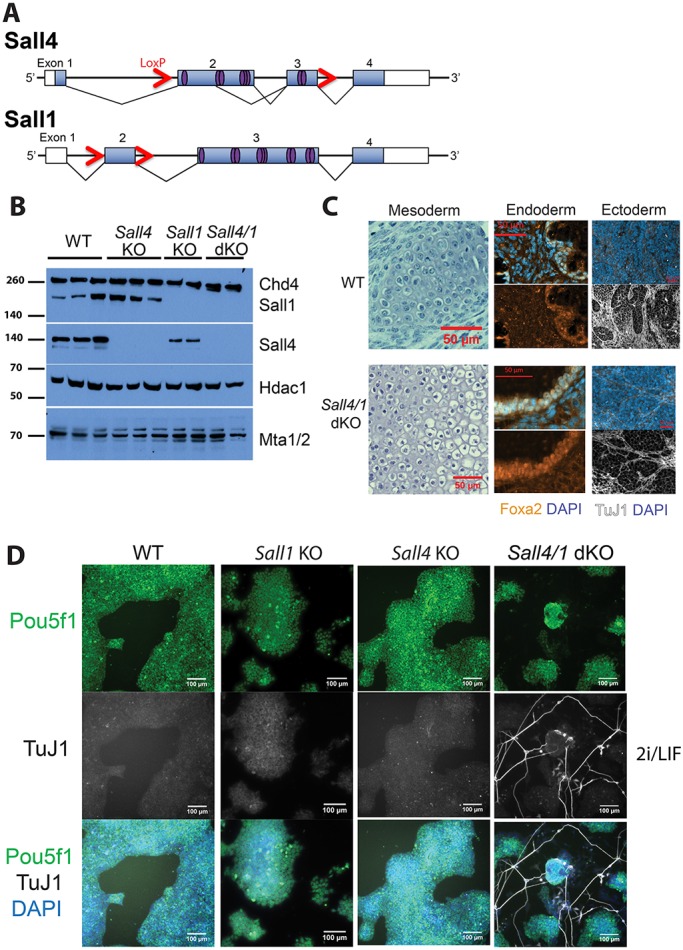
**Sall4 and Sall1 are dispensable for mouse ESC self-renewal.** (A) Schematic of targeted *Sall4* and *Sall1* genomic loci. Boxes represent exons, and filled boxes indicate the coding sequence; red arrows represent LoxP sites; purple ovals represent zinc-finger domains. (B) Western blot of wild-type (WT), *Sall4* null (hereafter *Sall4* KO), *Sall1* null (hereafter *Sall1* KO) and *Sall4/1* double-null (hereafter *Sall4/1* dKO) ESC lines in 2i/LIF. The blot was probed with anti-Chd4, anti-Sall1, anti-Sall4, anti-Hdac1 and anti-Mta1/2 antibodies. Molecular weights are shown at left in kDa. Note that the *Sall1* KO line is heterozygous for *Sall4* (*Sall1*^−/−^*; Sall4^flox/^*^−^). (C) Representative images from teratoma assays of mesoderm, endoderm and ectoderm tissues derived from WT (top) and *Sall4/1* dKO. Cartilage tissue is shown for mesoderm by H&E staining, immunofluorescence staining for Foxa2 (with DAPI) for endoderm, and for TuJ1 (with DAPI) for ectoderm. (D) Immunofluorescence of WT, *Sall1* KO, *Sall4* KO and *Sall4/1* dKO ESCs grown in 2i/LIF, stained for Pou5f1 (green), TuJ1 (white) and with DAPI (blue). Out of all DAPI-stained *Sall4/1* dKO ESCs per field, 2.09±1.19 (mean±s.d.) also stained positively for TuJ1. Six images were used to generate counts. Scale bars: 50 µm in C; 100 µm in D.

To deﬁne the function of Sall4 in pluripotent cells, ESCs were made homozygous for a previously described *Sall4* conditional allele (Sakaki-Yumoto et al., 2006) by two different methods: gene targeting and derivation from homozygous *Sall4* floxed mice followed by Cre-mediated recombination. The *Sall4* null ESC lines lack exons two and three, which contain all of the zinc-ﬁnger domains found in Sall4 (Fig. 1A). Although a truncated *Sall4* transcript is produced from this allele, no protein is detectable (Fig. S1A,B). To rule out potential compensation by the related Sall1 protein (Yuri et al., 2009), which is the only other Sall protein expressed in wild-type (WT) ESCs (Fig. S1A), we also derived ESCs from *Sall1^flox/flox^; Sall4^flox/flox^* mice. These cells were then used to obtain *Sall1^−/−^; Sall4^flox/−^* (referred to as *Sall1* null) and *Sall1^−/−^; Sall4^−/−^* (referred to as *Sall4/1* double-null) ESC lines (Fig. 1A,B) after Cre transfection and clonal isolation. Deletion of either *Sall1* or *Sall4* had no effect on the transcription level of the other gene (Fig. S1A). *Sall1* null, *Sall4* null, and *Sall4/1* double-null ESCs were viable and were able to be maintained as self-renewing cultures in 2i/LIF conditions (Fig. S1C). All ESC lines tested (WT, *Sall1* null, *Sall4* null and *Sall4/1* double-null cells) were able to give rise to tissues representing all three germ layers in teratoma assays, indicating that Sall4 and Sall1 are dispensable for ESC potency (Fig. 1C).

Although loss of both Sall1 and Sall4 was compatible with self-renewal in 2i/LIF conditions, there was considerably more spontaneous differentiation in double-mutant cultures than with either single mutant. The *Sall4/1* double-null differentiated cells present in 2i/LIF cultures sent out long processes that stained positively for the neuronal marker TuJ1 (also known as Tubb3), indicative of postmitotic neurons (Fig. 1D). When plated into serum/LIF conditions (in the absence of feeders), both *Sall4* null and *Sall4/1* double-null cells showed widespread differentiation (Fig. S1D). By contrast, *Sall1* null cells behaved similarly to WT in all conditions tested in this study.

**Fig. 2.**
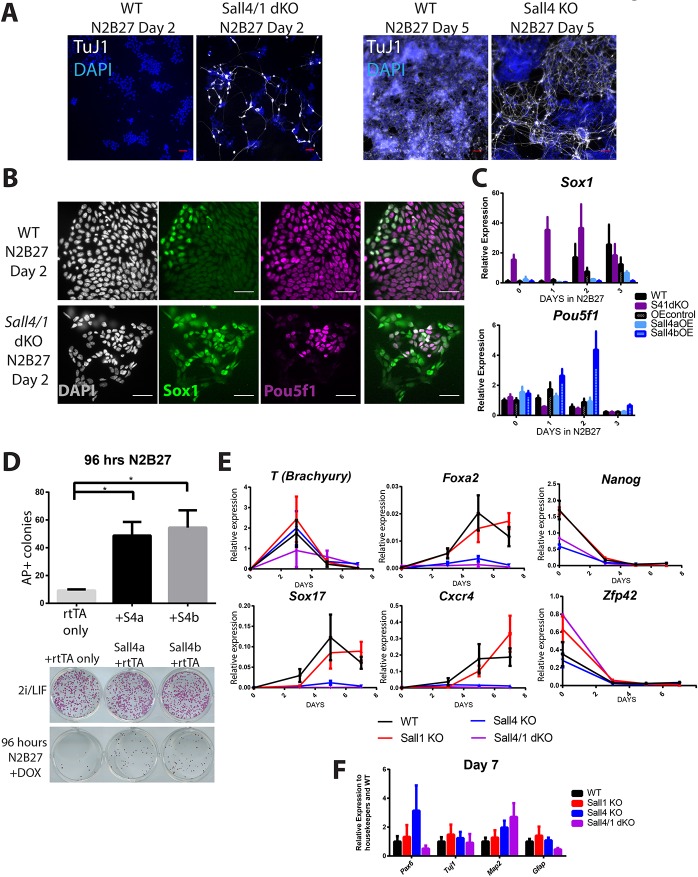
**Sall4 and Sall1 block neural differentiation.** (A) Representative immunofluorescence images of WT and *Sall4/1* dKO cells after 2 days in N2B27 (left), or WT and *Sall4* KO after 5 days in N2B27 (right) stained for TuJ1 (white) and with DAPI (blue). (B) Representative immunofluorescence images of WT and *Sall4/1* dKO cells after 2 days in N2B27, stained with DAPI (white) or for Sox1 (green) and Pou5f1 (magenta). The right-hand image is a composite of Pou5f1 and Sox1. (C) Expression of *Pou5f1* and *Sox1* in WT ESCs, *Sall4/1* (S41) dKO ESCs, ESCs overexpressing (OE) Sall4a or Sall4b and their control at day 0, 1, 2 and 3 in N2B27 was measured by qRT-PCR. OE control refers to WT cells expressing the Tet-transactivating factor only. OE control and Sall4 OE cell lines were cultured in doxycycline (DOX) for the entire timecourse. Expression is plotted relative to housekeeping genes as well as to their respective WT controls. Error bars represent s.e.m. between replicates (*N*=3-5). (D) Alkaline phosphatase (AP) assay of Tet-inducible Sall4 OE cell lines. rtTA refers to the Tet-transactivating factor. Cells were cultured for 96 h in N2B27+DOX (or maintained in 2i/LIF conditions as a control) before replating in 2i/LIF conditions for 5 days. Mean number of AP-positive colonies is shown. Error bars represent s.e.m. (*N*=4); *P≤0.05, one-way ANOVA followed by Dunnett's multiple comparisons test. Shown below is an example of colonies produced by the indicated ESC lines either with (below) or without (above) 96 h in differentiation conditions. (E) Gene expression analysis across the endoderm differentiation timecourse for the indicated genes in WT, *Sall1* KO, *Sall4* KO and *Sall4/1* dKO cells. Error bars represent s.e.m. between replicates (*N*=3-9). (F) Gene expression analysis at day 7 of the endoderm differentiation protocol in WT, *Sall1* KO, *Sall4* KO and *Sall4/1* dKO cells. The data are plotted relative to the WT samples. Error bars represent s.e.m. between replicates (*N*=3-9). Scale bars: 50 µm.

These observations suggested that Sall4 and Sall1 are involved in suppressing neural differentiation in ESCs. To test this hypothesis, single- and double-mutant cultures were subjected to a standard neuroectodermal differentiation protocol (Ying et al., 2003). Whereas WT cultures did not produce TuJ1-expressing neurons during the first 5 days of this protocol, TuJ1-expressing cells displaying neuronal morphology could clearly be seen by day 5 in *Sall4* null cultures and by day 2 in the *Sall4/1* double-null cultures (Fig. 2A). After only 2 days of the protocol, the majority of *Sall4/1* double-null cells had activated expression of the neural progenitor marker Sox1, and many had extinguished Pou5f1 expression, whereas Pou5f1 was still ubiquitously expressed in WT cells at this point and only a few WT cells had activated Sox1 (Fig. 2B,C). Thus, absence of Sall proteins in ESCs results in an accelerated pace of ESC exit from self-renewal and entry into the neural differentiation pathway.

As loss of Sall4 is associated with accelerated differentiation, we predicted that overexpression of Sall4 should result in reduced ESC differentiation. To test this hypothesis, cDNAs encoding Sall4a and Sall4b were expressed either singly or together in a doxycycline-inducible system in WT ESCs (Fig. S1F,G). The Sall4-overexpressing ESCs were then grown in differentiation conditions for 96 h, prior to plating back into 2i/LIF conditions. WT cells expressing the doxycycline-inducible transactivator, but no cDNAs, produced very few alkaline phosphatase-positive colonies after this procedure, indicating that most had undergone lineage commitment (Fig. 2D). By contrast, ESCs overexpressing Sall4 isoforms, either singly or together, produced an increased number of alkaline phosphatase-positive colonies, indicating that overexpression of Sall4 interferes with lineage commitment in ESCs. Further, ESCs overexpressing Sall4 proteins showed persistent *Pou5f1* expression and reduced *Sox1* expression in the neural differentiation timecourse (Fig. 2C; Fig. S1F). Together, these experiments demonstrate that Sall proteins act to slow the pace of neural differentiation in ESC cultures.

To test whether the Sall proteins act as general differentiation inhibitors in ESCs, we next assessed the ability of *Sall4* and *Sall4/1* mutant ESCs to differentiate towards a definitive endoderm fate (Morrison et al., 2008). Although mutant cells were able to silence pluripotency markers and to activate expression of brachyury (*T*), they subsequently failed to activate the endoderm markers *Sox17*, *Foxa2* and *Cxcr4* (Fig. 2E), but neither did they show evidence for having activated a neural programme (Fig. 2F). The failure of *Sall4* null and *Sall4/1* double-null ESCs to adopt either an endodermal or neural fate in this differentiation protocol indicates that Sall4 and Sall1 are not general differentiation inhibitors in ESCs.

### Sall4 and Sall1 prevent inappropriate activation of neural genes in ESCs, but are not required for maintenance of the pluripotency GRN

**Fig. 3.**
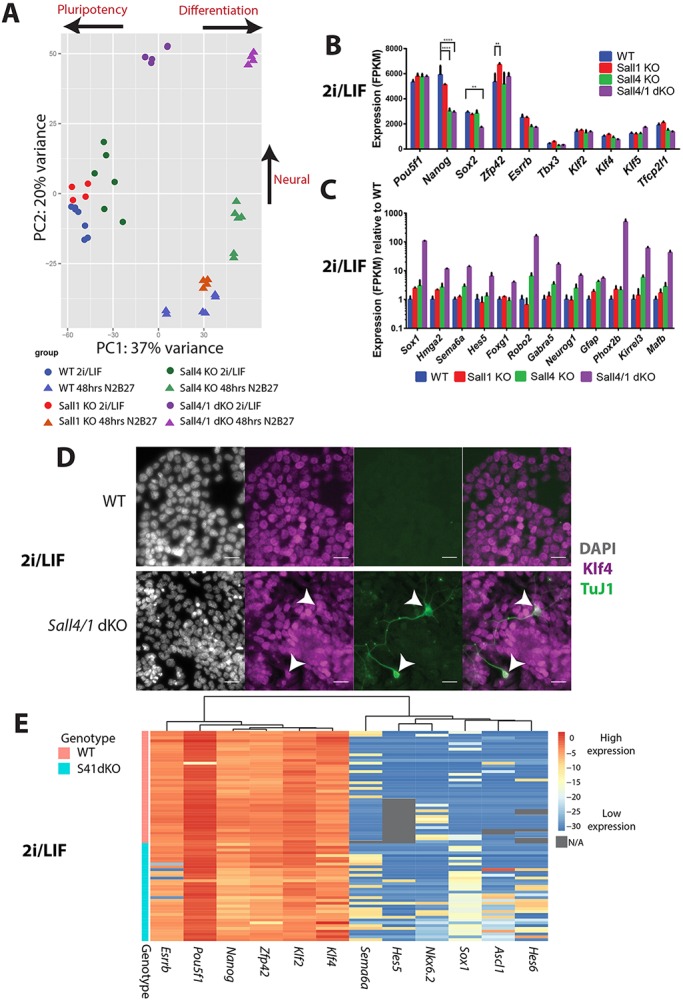
**Sall4** **and Sall1 prevent activation of the neurogenesis transcriptional programme but are dispensable for maintaining the pluripotency network.** (A) PCA plot representing RNA-seq data from WT, *Sall1* KO, *Sall4* KO and *Sall4/1* dKO cells in self-renewing (2i/LIF) or differentiation (48 h N2B27) culture conditions. Each point represents a separate biological replicate and each genotype is represented by two to three independent cell lines. (B) FPKM values from RNA-seq analysis showing expression of the indicated genes in WT, *Sall1* KO, *Sall4* KO and *Sall4/1* dKO cells in 2i/LIF. Error bars represent s.d. *N*=4-6 from two to three independent cell lines. **P≤0.01, ****P≤0.0001, two-way ANOVA followed by a Dunnett's multiple comparison test. (C) Expression of example neural genes is significantly upregulated (see supplementary Materials and Methods) in *Sall4/1* dKO compared with WT cells in 2i/LIF conditions. FPKM values relative to WT levels are shown for all cells. *N*=4-6 from two to three independent cell lines. Error bars represent s.d. (D) Example immunofluorescence images of WT and *Sall4/1* dKO cells in 2i/LIF stained with DAPI (white) and for Klf4 (magenta) and TuJ1 (green). The right-hand image is a composite of Klf4 and TuJ1. Arrowheads indicate TuJ1-positive cells that co-express Klf4. Scale bars: 25 µm. (E) Heat map constructed from single ESC expression data based on hierarchical clustering for pluripotency-associated genes (*Esrrb*, *Pou5f1*, *Nanog*, *Zfp42*, *Klf2* and *Klf4*) and neural-associated genes (*Sema6a*, *Hes5*, *Nkx6.1*, *Sox1*, *Ascl1* and *Hes6*). Individual cells are ordered from top to bottom: the top 40 are WT cells and the bottom 35 are *Sall4/1* dKO cells. Normalised Ct values (key on the right) refer to –ΔCt values normalised to housekeeping genes (*Atp5a1*, *Ppia* and *Gapdh*). Grey boxes indicate that data are not available (N/A).

Sall proteins are known to be transcriptional regulators, so we suspected that they would limit neural differentiation by controlling gene expression. To identify the Sall4- and Sall1-dependent transcriptional programmes during ESC self-renewal and during early stages of neural differentiation, we measured global gene expression profiles by RNA-seq in WT, *Sall1* null, *Sall4* null and *Sall4/1* double-null ESCs in self-renewing conditions (2i/LIF) and after 48 h in differentiation conditions (N2B27) (Table S1). Global gene expression profiles of WT, *Sall1* null and *Sall4* null ESCs are largely similar in 2i/LIF conditions, resulting in replicates of these genotypes clustering loosely together on the left-hand side of a principal component analysis (PCA) plot (Fig. 3A). By contrast, the double nulls show a distinct profile in the upper middle section of the plot, consistent with increased expression of neural differentiation markers (Fig. S2A) and the presence of morphologically neural cells in 2i/LIF cultures of *Sall4/1* double-null cells (Fig. 1D). After 48 h in differentiation conditions (N2B27) the WT and *Sall1* null ESCs show a similar change in gene expression profiles, moving to the lower right portion of the plot consistent with silencing of pluripotency markers and activation of early differentiation markers (Fig. S2A). *Sall4* null ESCs occupy a somewhat distinct location, presumably owing to partial activation of a neural GRN (Fig. 3A; Fig. S2A). *Sall4/1* double-null cells in N2B27 conditions remain at the top of the plot but move even further to the right, consistent with more complete adoption of a neural phenotype (Fig. S2A).

The majority of genes found to be misexpressed in either *Sall1* or *Sall4* null ESCs are also misexpressed in *Sall4/1* double-null ESCs, and there is a strong correlation in the direction of the change (Fig. S2B,C). Genes showing increased expression in *Sall4* null or *Sall4/1* null cells show very high enrichment for Gene Ontology (GO) terms involving development, including ‘neurogenesis' and ‘nervous system development' (Fig. S2D). Further, 42% of genes normally upregulated in WT cells after 48 h in N2B27 are already upregulated in *Sall4/1* double-null cells in 2i/LIF, and the top GO term associated with this group of genes is ‘nervous system development' (Fig. S2E). This further supports the hypothesis that Sall4 and Sall1 act together to prevent activation of a neural gene expression programme in ESCs.

Sall4 has been reported to be a component of the pluripotency network, i.e. playing some role in maintaining the GRN underpinning the pluripotent state (Dunn et al., 2014; van den Berg et al., 2010). Findings from the analysis of expression data for individual genes are inconsistent with such a role. Fig. 3B shows that the expression level of many pluripotency-associated genes in ESCs is not significantly altered in the absence of Sall4 and/or Sall1. Although *Sall4* null and *Sall4/1* double-null ESCs show a reduction in *Nanog* expression, and *Sall4/1* double mutants also show a reduction in levels of *Sox2*, this reduction does not result in destabilisation of expression levels of the other pluripotency-associated genes in 2i/LIF.

Although expression of pluripotency markers is largely normal, *Sall4/1* double-null ESC cultures in 2i/LIF conditions expressed elevated levels of genes associated with neuronal differentiation (Table S1; a subset is shown in Fig. 3C). Surprisingly, a fraction of the *Sall4/1* double-null cells in 2i/LIF conditions expressed markers of both a neural (TuJ1) and a pluripotent (Pou5f1 or Klf4) lineage (Fig. 1D, Fig. 3D). In order to expand on this observation we measured gene expression levels in individual ESCs by quantitative RT-PCR (qRT-PCR). As expected, WT ESCs maintained in 2i/LIF conditions robustly expressed pluripotency genes but rarely expressed neural genes (Fig. 3E). *Sall4/1* double-null ESCs showed increased expression of neural genes consistent with RNA-seq and qRT-PCR from bulk cell populations. In addition to aberrant expression of neural genes, individual *Sall4/1* double-null ESCs simultaneously maintained the expression of most pluripotency genes (Fig. 3E). This indicates that components of both the pluripotency and neural differentiation GRNs can be active simultaneously in individual *Sall4/1* double-mutant ESCs. We conclude that in ESCs Sall4 and Sall1 act to prevent activation of neural genes, but are dispensable for maintenance of the pluripotency GRN.

### Sall4 is an enhancer-binding protein that controls expression of developmental genes

We next sought to identify Sall4-bound genomic sequences in ESCs using ChIP-seq. Previous studies of Sall4 binding to the ESC genome used mouse microarrays (ChIP-Chip), the coverage of which is heavily biased towards genes and promoters, and therefore do not provide genome-wide coverage (Lim et al., 2008; Rao et al., 2010; Tanimura et al., 2013; Yang et al., 2008; Yuri et al., 2009). To facilitate immunoprecipitation of Sall4, the endogenous *Sall4* locus was targeted to add an epitope tag (Avi-3×FLAG) at the C-terminus of the protein (Fig. S3A,B). Immunoprecipitation with an anti-FLAG antibody verified that addition of the epitope tag did not interfere with its known interaction with the NuRD complex (Bode et al., 2016; Kloet et al., 2015; Yuri et al., 2009) (Fig. S3C) nor with its intracellular localisation (Fig. S3D). To verify that addition of the epitope tag did not interfere with normal Sall4 function, ESCs were produced in which both *Sall4* alleles were targeted with the epitope tag. These cells did not show accelerated neural differentiation like *Sall4* null ESCs, and were able to activate endodermal genes when subjected to the endodermal differentiation protocol, unlike *Sall4* null cells (Fig. S3E-G). Thus, addition of a C-terminal epitope tag did not detectably interfere with Sall4 function in ESCs.

**Fig. 4.**
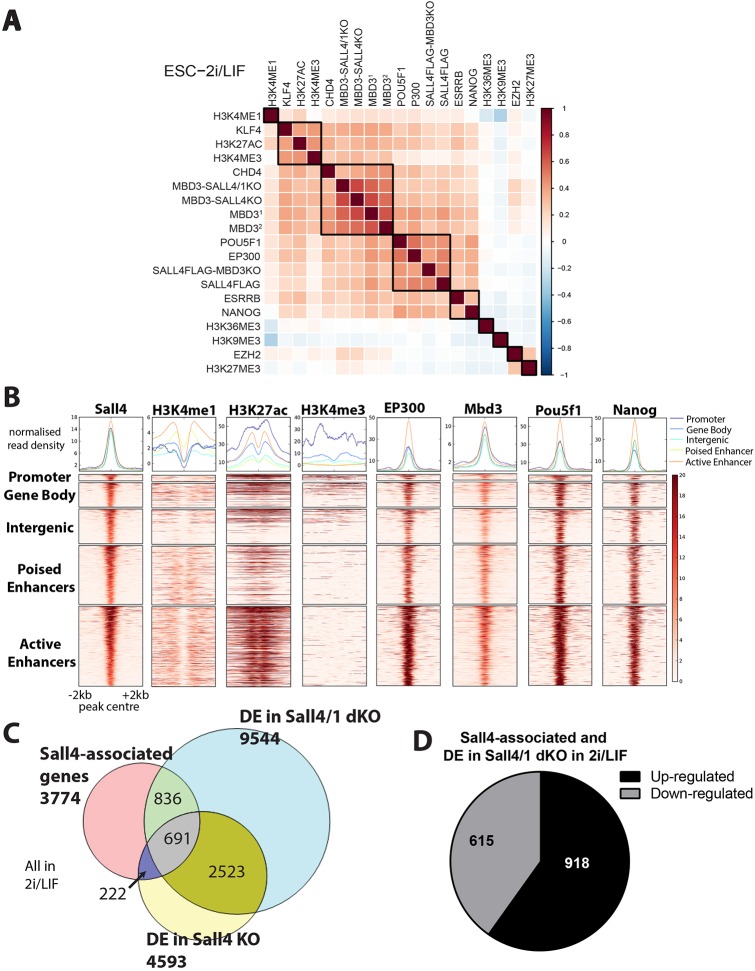
**Sall4 is an enhancer-binding protein and acts to activate as well as inhibit gene expression.** (A) Heat map showing correlation between ChIP peaks for the indicated histone modifications and transcription factors in 2i/LIF. ChIP datasets from mutant cells lines are labelled with the antibody used for the ChIP and then the cell line in which they were performed (i.e. MBD3-SALL4KO refers to Mbd3 Chip in *Sall4* KO cells). Mbd3^1^ indicates Mbd3 ChIP performed in the parent line of the *Sall4* KO and Mbd3^2^ indicates Mbd3 ChIP performed in the parent cell line of the *Sall4/1* dKO. Datasets used are listed in the supplementary Materials and Methods. (B) Heat maps of binding profiles of H3K4me1, H3K27ac, H3K4me3, Ep300, Mbd3, Pou5f1 and Nanog shown within 2 kb of the centre of Sall4 peaks. These are partitioned into five groups: promoter-, gene body-, intergenic-, poised enhancer- and active enhancer-associated peaks. Each category is defined in the supplementary Materials and Methods. Graphs above the heat maps show enrichment. (C) Venn diagram showing the overlap between Sall4-associated genes (pink), differentially expressed (DE) genes in *Sall4* KO versus WT (yellow) and differentially expressed genes in *Sall4/1* dKO compared with WT (blue). All in 2i/LIF. (D) Pie chart showing the Sall4-associated genes that are differentially expressed in *Sall4/1* dKO cells compared with WT cells, and the numbers that are upregulated or downregulated. All in 2i/LIF.

Hierarchical clustering of Sall4 ChIP-seq data along with data available in CODEX for a number of transcription factors and histone modifications in ESCs (Sanchez-Castillo et al., 2015) shows that the Sall4 binding profile is well correlated with those of pluripotency-associated transcription factors such as Nanog, Pou5f1, Esrrb and Klf4, as well as for the NuRD component proteins Mbd3 and Chd4 (Fig. 4A,B). Further positive correlation exists with marks of active chromatin H3K4me1, H3K27ac and the histone acetyltransferase Ep300, but not with H3K4me3, consistent with Sall4 associating predominantly with enhancer sequences. Sall4 binding does not correlate with a mark of transcribed gene bodies (H3K36me3), repressive chromatin marks (H3K27me3, H3K9me3) or with a component of the PRC2 complex (Ezh2).

To ascertain whether and how the repertoire of Sall4-bound sequences might explain the function of Sall4 in preventing neural differentiation, we assigned each Sall4 peak to its nearest gene. A large proportion of Sall4-associated genes are differentially expressed in the *Sall4/1* double nulls relative to WT cells in either 2i/LIF or N2B27 conditions (40.5% and 43%, respectively; Fig. 4C, Fig. S4A). The genes bound by Sall4 and inappropriately activated in *Sall4/1* double nulls are associated with GO terms involving development and neurogenesis (Fig. S4B), consistent with the crucial function of Sall4 in inhibiting neural specification being to prevent activation of neurogenesis genes in self-renewing conditions and during the early stages of differentiation. Globally, Sall4 is not only a transcriptional repressor, as ∼40% of Sall4-bound and differentially expressed genes show downregulation in the absence of Sall4 (Fig. 4D; Fig. S4C). Notably, several of the GO terms associated with these genes are also associated with genes showing downregulation in WT cells undergoing neural differentiation (Fig. S2E).

### NuRD is the major biochemical interactor of Sall4

**Fig. 5.**
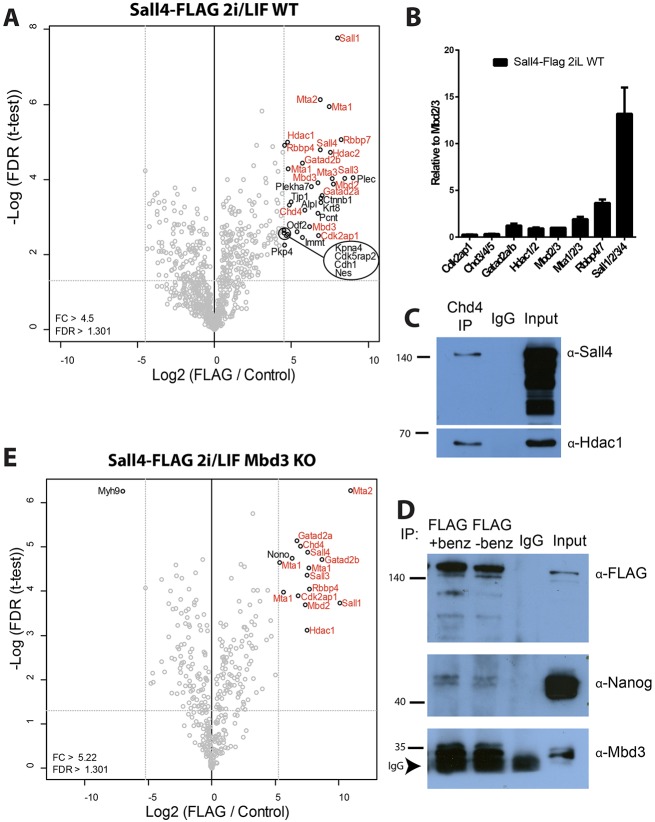
**Identification and stoichiometry of Sall4-interacting proteins in ESCs.** (A) Volcano plot showing the significant interactors of Sall4 (black circles) in WT ESCs cultured in 2i/LIF conditions. The proteins highlighted in red are known NuRD components. (B) Stoichiometry of NuRD components and Sall proteins relative to Mbd2/3. Error bars represent s.d. from three independent immunoprecipitations/mass spectrometry replicates. (C) Western blot of immunoprecipitation with anti-Chd4 antibody, IgG control, or 1/10 of input of nuclear extract from WT ESCs and probed with anti-Sall4 (top) or anti-Hdac1 (bottom) antibodies. The anti-Sall4 panel shows a long exposure to visualise the Sall4 band in the Chd4 immunoprecipitation (IP) lane, revealing multiple variously SUMOylated forms of Sall4 in the input lane. Numbers to the left indicate size markers in kDa. (D) Western blot of immunoprecipitation with anti-FLAG antibody, IgG control, or 1/10 of input of nuclear extract from Sall4-FLAG ESCs and probed with the antibodies indicated at right. The anti-FLAG immunoprecipitations are shown with and without the general nuclease benzonase, which makes no difference to the Nanog or Mbd3 association. Size markers are shown on the left in kDa. (E) Volcano plot showing the significant interactors of Sall4 (black circles) in *Mbd3* KO ESCs cultured in 2i/LIF conditions. The proteins highlighted in red are known NuRD components.

To better understand how Sall4 exerts its transcriptional regulatory activity, we identified Sall4-interacting proteins in the Sall4-FLAG ESC line using mass spectrometry. As expected, Sall4 robustly co-purified with the core components of the NuRD complex (Fig. 5A, indicated in red). A number of other interacting proteins are shown in Fig. 5A, one of which (Kpna4, an importin subunit) has previously been identified as a NuRD interactor (Kloet et al., 2015). The remainder are proteins normally found in the cytoplasm and/or centriole, whereas in ESCs we find that Sall4 is strictly a nuclear protein (Fig. S3D). Although consistent with the possibility of Sall4 interacting with a centrosome-associated NuRD complex (Sakai et al., 2002; Sillibourne et al., 2007), these were not considered further.

Sall4-FLAG was purified at ∼14-fold excess relative to NuRD [assuming one Mbd2/3 protein and one Sall4 protein per NuRD complex (Kloet et al., 2015)] (Fig. 5B). As we used extraction conditions previously shown to maintain Sall4-NuRD interactions (Kloet et al., 2015), and the Sall4 protein that we purified was expressed from its endogenous locus, this high ratio of Sall4 to NuRD cannot be dismissed as an artefact of protein overexpression or methodology. Immunoprecipitation of Chd4 from WT cells also recovers only a fraction of the Sall4 present in the nucleus, consistent with the majority of Sall4 not being bound to the NuRD complex (Fig. 5C). By contrast, Sall4 immunoprecipitation recovers a large proportion of Mbd3 present in the nucleus, which supports our assertion that the Sall4-NuRD interaction is not being lost due to technical reasons (Fig. 5D). Together, these data show that a relatively minor fraction (∼7%) of Sall4 interacts with the NuRD complex, whereas a large proportion of Mbd3-NuRD contains Sall4.

Sall4-FLAG purification was repeated in ESCs lacking Mbd3, a major structural NuRD component protein (Kaji et al., 2006; Reynolds et al., 2012), to identify NuRD-independent interactors of Sall4. Purification of Sall4 and associated proteins in *Mbd3* null ESCs again yielded NuRD components (but no Mbd3), which presumably derive from the small amount of Mbd2-NuRD present in these cells (Fig. 5E). In addition to NuRD components, the only significant interacting protein was the Non-POU domain-containing octamer-binding protein Nono, which was not identified in WT cells. Nono was purified at extremely low levels and is unlikely to be a significant interacting protein.

The pluripotency-associated factors Pou5f1 and Nanog have previously been reported to co-purify with overexpressed Sall4 protein in ESCs grown in serum/LIF conditions (van den Berg et al., 2010), and the endogenous proteins have been shown to interact by immunoprecipitation (Rao et al., 2010; van den Berg et al., 2010; Wu et al., 2006). Although we did identify Pou5f1 peptides in our experiment, these were very few and far below significance (Fig. S5A). Although no Nanog peptides were identified in our mass spectrometry experiments, we were able to detect an interaction between Sall4 and Nanog protein by immunoprecipitation of our tagged Sall4 and western blotting (Fig. 5D). Only a very small proportion of the endogenous nuclear Nanog protein was found to associate with Sall4, which presumably represents a weak and/or infrequent interaction that is below the minimum threshold of detection for mass spectrometry with endogenous Sall4.

We conclude that an interaction between Sall4 protein and pluripotency factors is detectable, and may rise above background in mass spectrometry experiments using overexpressed Sall4 protein, but involves a very small proportion of total endogenous Sall4 protein and thus does not represent a major interaction. Thus, although ∼7% of Sall4 protein is found within the NuRD complex, the majority of Sall4 protein in ESCs does not appear to stably associate with any other protein, but may associate transiently or infrequently with pluripotency-associated transcription factors.

### Sall4 neither recruits nor functions through the NuRD complex

**Fig. 6.**
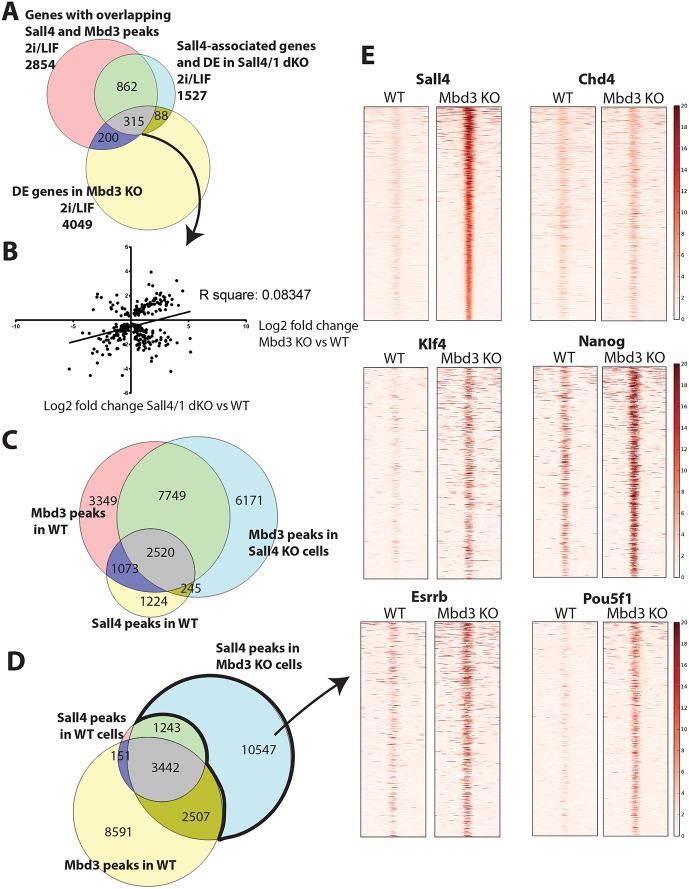
**Sall4 neither recruits nor acts through the NuRD complex.** (A) Venn diagram showing the overlap between genes associated with overlapping Sall4 and Mbd3 peaks in 2i/LIF (pink), genes associated with a Sall4 peak and differentially expressed in *Sall4/1* dKO in 2i/LIF (blue), and genes differentially expressed in *Mbd3* KO cells in 2i/LIF (yellow). (B) Plot comparing the log_2_ fold change between differentially expressed genes in both *Sall4/1* dKO and *Mbd3* KO cells compared with WT cells. These genes have Sall4 and Mbd3 overlapping peaks and are differentially expressed in *Sall4/1* dKO cells as well as in *Mbd3* KO cells compared with WT, all in 2i/LIF (i.e. the 315 genes shown in grey in A). A linear regression was performed to generate the R-square value. (C) Venn diagram showing overlap between Mbd3 peaks in WT cells (pink), Mbd3 peaks in *Sall4* KO cells (blue) and Sall4 peaks in WT cells (yellow). The WT used for this comparison is the parent line of the *Sall4* KO cells. All are in 2i/LIF. (D) Venn diagram showing the overlap of Sall4 peaks in WT cells (pink) with Sall4 peaks in *Mbd3* KO cells (blue) and Mbd3 peaks in WT cells (yellow). (E) ChIP-seq heat maps of 2 kb either side of the Sall4 peaks only found in *Mbd3* KO ESCs and not normally bound by Mbd3 (bold black outline in D) for the indicated transcription factors in WT and *Mbd3* KO ESCs.

Given that a large proportion of NuRD contains Sall4, and that Sall4 and Mbd3 co-occupy a number of genomic sites, the standard model of transcription factor–co-repressor interaction stipulates that Sall4 should recruit NuRD to effect transcriptional repression. If this were true, we would expect that Sall4- and Mbd3-associated genes should show similar changes in expression in *Sall4/1* double-null ESCs as in *Mbd3* null ESCs. Of all Sall4-bound genes showing differential expression in *Sall4/1* double-null ESCs, 20% (315 of 1527 genes) were also bound by Mbd3 and showed transcriptional changes in *Mbd3* null ESCs (Fig. 6A). There is no correlation (neither positive nor negative) in the direction of gene expression changes between *Mbd3* null and *Sall4/1* double-null cells for these 315 genes (Fig. 6B), making it very unlikely that they are co-regulated by Sall4 and NuRD. Similarly, those genes misexpressed in *Sall4/1* or *Mbd3* mutant cells in differentiation conditions (N2B27) show no correlation in terms of the direction of gene expression change (Fig. S6B). Therefore, our analysis provides no evidence that Sall4 and NuRD act in concert to regulate gene expression.

If Sall4 acts to recruit NuRD to specific sites, then we would expect that many Mbd3- and Sall4-bound regions would show loss of Mbd3 binding in *Sall4* null ESCs. Of 4422 Mbd3 peaks lost in *Sall4* null cells, 24% (1073) were bound by Sall4 in WT cells, while 20% of the Mbd3 peaks lost in *Sall4/1* double-null cells were Sall4-bound sites (Fig. 6C; Fig. S6C). This amounts to 7.3% of all Mbd3 sites that could be recruited by Sall4, corresponding to less than 5% of genes misregulated in the *Mbd3* nulls, yet the transcriptional changes seen at these genes in *Mbd3* null ESCs do not correlate with those seen in *Sall4/1* double-null cells (Fig. S6D). If the same analysis is performed using a less stringent method of defining peaks from ChIP replicates (i.e. by merging replicates rather than using the IDR method; see Materials and Methods), then 3.0% of Mbd3 peaks show both Sall4 dependency and Sall4 binding (Fig. S6E). Thus, we find no evidence to support a model whereby Sall4 directs the recruitment of NuRD to control gene expression in ESCs.

### Sall4 occupies enhancers with pluripotency-associated transcription factors to regulate transcription

Although Sall4 does not dictate NuRD chromatin targets, surprisingly, NuRD was found to influence the genome-wide distribution of Sall4. ChIP-seq for Sall4-FLAG in *Mbd3* null cells identified 3.5-fold more Sall4-bound locations than in WT cells (17,739 versus 5062; Fig. 6D). The Sall4-bound sites found only in *Mbd3* null cells predominantly consisted of enhancers, as is seen for the WT cohort of Sall4-bound sites (Fig. S6F). In addition to Sall4 binding to novel sites in the absence of Mbd3/NuRD (e.g. *Tex13* and *Ppp2r2c* enhancers; Fig. S7A,B), Sall4 also shows increased binding at some peaks seen in WT cells (e.g. *Nanog*, but not *Pou5f1*; Fig. S7A,B). This indicates that more Sall4 protein is available to bind chromatin in the absence of Mbd3. Indeed, *Mbd3* null ESCs contain moderately (2- to 3-fold) increased levels of Sall4 protein, despite there being no increase in *Sall4* transcript levels (Table S1, Fig. S5B).

By focusing on the Sall4-enriched regions seen only in *Mbd3* null ESCs, we were able to investigate the consequences of novel Sall4 binding to enhancer sequences. In *Mbd3* null cells, they not only gain Sall4 protein enrichment but also become enriched for the pluripotency-associated transcription factors Pou5f1, Nanog, Klf4 and Esrrb (Fig. 6E). Notably, no increase in Chd4 protein enrichment is seen at these same sites in the *Mbd3* null cells, indicating that the observed increase in transcription factor association is not simply a consequence of these sites becoming generally more accessible. What consequence does recruitment of transcription factors have on these enhancers? Assigning these sites to their nearest genes identifies 6666 genes, of which nearly one-fifth (1166) show a significant gene expression change in *Mbd3* null ESCs (Fig. S7C), with approximately equal numbers showing increased or decreased expression (Fig. S7D). These sites are not associated with significant Mbd3 enrichment in WT cells (Fig. 6D), yet they account for nearly one-third (1166/4049) of all genes misexpressed in *Mbd3* null ESCs (Fig. S7C).

**Fig. 7.**
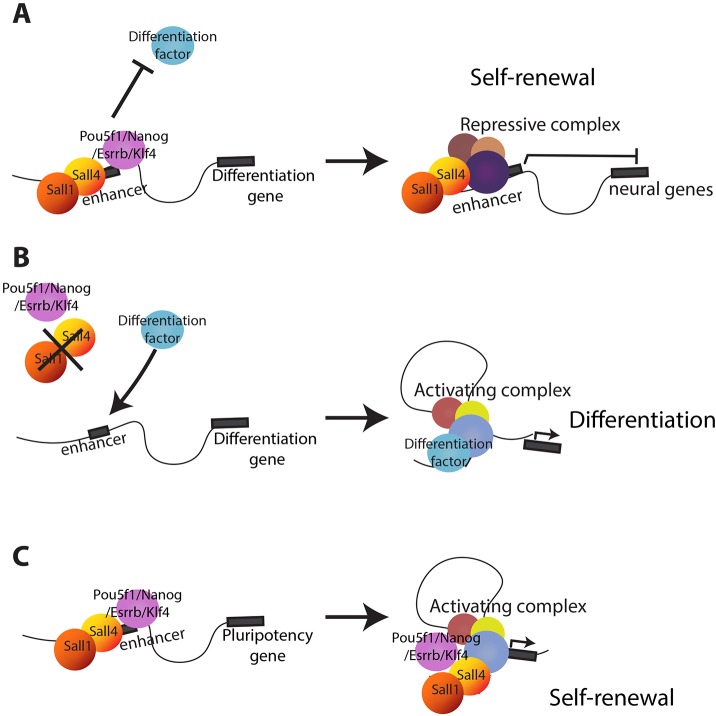
**Model of Sall4 activity in ESCs.** (A) Sall4 binds to the enhancer of a gene normally expressed during neural development along with Nanog, Pou5f1, Klf4 and Esrrb, preventing the association of a lineage-specific transcription factor (differentiation factor). The result is failure to activate the neural gene. (B) In the absence of Sall4 and Sall1 the differentiation factor is no longer prevented from binding to the enhancer and the neural gene is then inappropriately activated. (C) At enhancers of genes normally expressed in undifferentiated ESCs the binding of Sall proteins along with Pou5f1, Nanog, Klf4 and Esrrb maintains transcriptional activation of the self-renewal gene.

Recruitment of Sall4 and four pluripotency-associated transcription factors to these enhancers is equally likely to result in gene activation as it is in repression. GO terms associated with upregulated genes involve development and motility (Fig. S7E), whereas genes showing decreased expression do not significantly associate with any specific GO term. Thus, enhancers able to increase transcription in response to the recruitment of this group of transcription factors are predominantly associated with developmental genes, whereas enhancers associated with other kinds of genes are not activated by these transcription factors, and indeed this recruitment interferes with transcription. In summary, we propose that Sall4 acts to prevent neural differentiation of ESCs by binding to enhancers along with other pluripotency-associated transcription factors, where their presence interferes with gene activation (Fig. 7). At other enhancers, binding of this same cohort of transcription factors increases transcription.

## DISCUSSION

Sall4 is an essential protein for early mammalian development. Here, we show that Sall4 and Sall1 function to prevent activation of neural development genes in ESCs, but are not required to maintain the pluripotent state. Sall4 is predominantly an enhancer-binding protein and, although it binds to a similar array of genomic locations as the pluripotency-associated proteins Pou5f1, Nanog, Esrrb and Klf4, it does not stably associate with these proteins. We further clarify the nature of the relationship between Sall4 and the NuRD complex. Although a proportion of Sall4 protein does stably interact with the NuRD complex, contrary to the standard model of co-repressor recruitment to DNA, Sall4 neither recruits NuRD to specific sites on DNA nor does it use NuRD to control expression of its target genes. Rather, Sall4 occupancy of enhancer sequences, along with other pluripotency-associated transcription factors, can either enhance or interfere with transcription, depending upon the target gene (Fig. 7).

Previous studies of Sall4 function in ESCs have produced conflicting conclusions about the role of Sall4 in ESC self-renewal (Rao et al., 2010; Sakaki-Yumoto et al., 2006; Tsubooka et al., 2009; Yuri et al., 2009; Zhang et al., 2006). It is very likely that the differing results obtained from these various laboratories are heavily influenced by the different culture conditions. Using a fully defined culture system [2i/LIF (Ying et al., 2008)] we show that Sall4 and Sall1 are dispensable for ESC self-renewal, but that they prevent premature activation of neural genes. This result agrees with a report that knockdown of Sall4 in 2i/LIF conditions does not significantly compromise ESC self-renewal (Dunn et al., 2014), and with our finding that Sall4 overexpression in WT cells inhibits neural differentiation (Fig. 2C). Notably, Yuri et al. (2009) were able to establish Sall4/1-double-knockout ESCs in serum/LIF conditions, indicating that this is not a difference in Sall4 function between different culture conditions. We show clearly that Sall4 is not an essential pluripotency factor, but rather is a differentiation inhibitor. We speculate that interference with Sall protein activity might enhance the efficiency of directed pluripotent cell neural differentiation protocols for disease modelling or regenerative medicine applications.

Using mass spectrometry on immunoprecipitated endogenous Sall4 protein, we find that ∼7% of nuclear Sall4 protein interacts with the NuRD complex. The simplest interpretation of this would be that this subset of Sall4 indirectly influences gene expression by recruiting NuRD to specific sequences. The problem with this scenario is that Sall4 and NuRD serve opposing functions in ESCs: NuRD facilitates exit of ESCs from the self-renewing state by restricting expression levels of pluripotency-associated genes (Reynolds et al., 2012), whereas Sall4 acts to prevent activation of neural genes and precocious neural specification in ESCs (Fig. 2). This is not what one would expect if NuRD collaborates with Sall4 to regulate the expression of Sall4 target genes. We find no evidence that Sall4 plays any significant role in recruiting NuRD to chromatin, nor that the expression levels of Sall4 target genes are sensitive to the presence or absence of NuRD.

We identified neither Nanog nor Pou5f1 as a significant Sall4-interacting protein in our proteomics experiments, although a weak interaction could be detected by immunoprecipitation and western blotting (Fig. 5D). Both of these proteins have been identified as Sall4 interactors in other studies (Rao et al., 2010; van den Berg et al., 2010; Wu et al., 2006). Our study differs from previous studies of Sall4 interactors as we have incorporated an epitope tag to the endogenous *Sall4* locus, and therefore have not introduced an extra copy of *Sall4* into ESCs. Sall4 dosage is important in somatic tissues (Koshiba-Takeuchi et al., 2006; Sakaki-Yumoto et al., 2006), so introduction of more Sall4, even if expressed at levels comparable to endogenous protein, would increase the concentration of nuclear Sall4 and might enable association with proteins such as Pou5f1 and Nanog (Fig. 7). Our results do not preclude an interaction between Sall4 and these pluripotency factors, but rather suggest that such interactions are either transient or involve only a minor fraction of total Sall4.

The Sall4 protein does not appear to have any enzymatic activity, does not recruit the NuRD complex to its sites of action, and does not have any other major, stably interacting proteins. Sall4 is predominantly found at enhancers, which are also often bound by pluripotency-associated transcription factors such as Pou5f1, Nanog, Klf4 and Esrrb (Fig. 4A,B, Fig. 7). Loss of Sall4 results in increased transcription of some Sall4-associated genes and reduced transcription of others, indicating that the outcome of Sall4 activity depends upon the sequence to which it binds (Fig. 7). We propose that accumulation of these transcription factors at enhancers that normally respond to lineage-specific transcription factors interferes with their activation, possibly by steric hindrance of transcription factor binding (Fig. 7A). In cells lacking the Sall proteins, this accumulation of transcription factors at neural genes does not occur and permits gene activation (Fig. 7B). By contrast, binding of these proteins to enhancers of genes normally expressed during ESC self-renewal promotes or enforces active transcription (Fig. 7C), although maintaining expression of pluripotency-associated genes does not strictly require the presence of Sall4. This scenario is similar to that seen for Pou5f1 during reprogramming, where Pou5f1 binding to enhancers of somatic genes in mouse embryonic fibroblasts correlates with transcriptional silencing of the associated gene, whereas Pou5f1 binding to enhancers of genes normally expressed in pluripotent cells correlates with activation (Chen et al., 2016). This would also mean that the dosage of Sall4 would be very important: too little Sall4 and some genes might be activated inappropriately, while too much Sall4 could interfere with the expression of lineage-appropriate genes. This could explain the observed haploinsufficiency of Sall4 during mammalian development (Koshiba-Takeuchi et al., 2006; Sakaki-Yumoto et al., 2006).

## MATERIALS AND METHODS

### Mouse ESC lines, culture and manipulation

All ESC lines were cultured in 2i/LIF conditions on gelatin-coated plates. ESC derivations were performed in 2i/LIF conditions. Gene targeting was carried out using homologous recombination methods and verified by long-range PCR, RT-PCR and western blotting. For details, including the antibodies used, see the supplementary Materials and Methods. Doxycycline treatment with alkaline phosphatase staining, the neural differentiation protocol and teratoma assay were performed as detailed in the supplementary Materials and Methods. All animal experiments were approved by the Animal Welfare and Ethical Review Body of the University of Cambridge and carried out under appropriate UK Home Office licenses.

### Chromatin immunoprecipitation and sequencing

ChIP-seq in ESCs was performed as previously described (Reynolds et al., 2012). For details, including the antibodies used, see the supplementary Materials and Methods.

### Bioinformatic analyses

Sall4-FLAG, Mbd3 and Chd4 ChIP-seq data were analysed using the irreproducible discovery rate (IDR) method, which assesses replicate agreement and therefore only calls peaks that are strong in all replicates (Landt et al., 2012; Li et al., 2011). This has the effect of removing false positives, but also of removing many weaker true positives. Thus, the set of ‘bound’ peaks used in the subsequent analyses is not comprehensive, but is of very high confidence and will represent only the strongest-bound peaks. Differentially expressed genes are listed in Table S1, and genes closest to Sall4 and Mbd3 peaks are listed in Table S2. For full details, see the supplementary Materials and Methods.

### Mass spectrometry

To identify Sall4 interactors, tryptic peptides obtained from affinity-purified nuclear proteins were subject to mass spectrometry analysis and LFQ peptide identification as described in the supplementary Materials and Methods.

### qRT-PCR

Single-cell expression analysis of pluripotency and lineage markers was performed by qRT-PCR using the TaqMan primers described in the supplementary Materials and Methods.
